# Supplementary material for: Interpretable Fine‐Grained Phenotypes of Subcellular Dynamics via Unsupervised Deep Learning
Source: Adv Sci (Weinh). 2024 Sep 6;11(41):2403547. doi: 10.1002/advs.202403547 (PMC11538677; doi:10.1002/advs.202403547)
Supplement: Supplementary file 1 — Supporting Information [file ADVS-11-2403547-s001.pdf]

ADVANCED SCIENCE
Open Access

Supporting Information

for *Adv. Sci.*, DOI 10.1002/advs.202403547

Interpretable Fine-Grained Phenotypes of Subcellular Dynamics via Unsupervised Deep Learning

*Chuangqi Wang, Hee June Choi, Lucy Woodbury and Kwonmoo Lee**

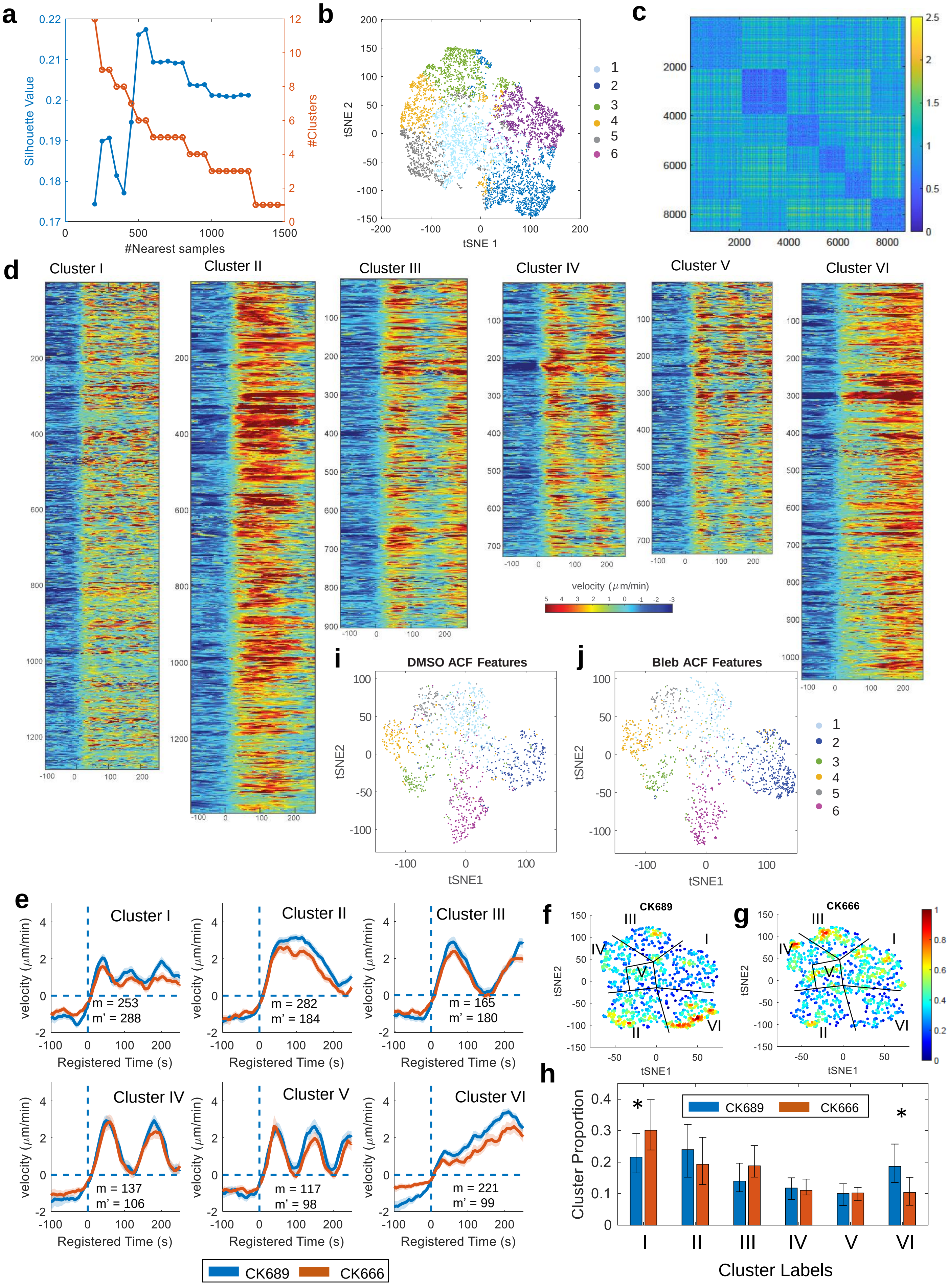

a
Silhouette Value
#Clusters
#Nearest samples
b
tSNE 1
tSNE 2
1
2
3
4
5
6
c
d
Cluster I
Cluster II
Cluster III
Cluster IV
Cluster V
Cluster VI
velocity (μm/min)
i
DMSO ACF Features
j
Bleb ACF Features
tSNE1
tSNE2
e
Cluster I
m = 253
m' = 288
Cluster II
m = 282
m' = 184
Cluster III
m = 165
m' = 180
Cluster IV
m = 137
m' = 106
Cluster V
m = 117
m' = 98
Cluster VI
m = 221
m' = 99
velocity (μm/min)
Registered Time (s)
CK689
CK666
f
CK689
g
CK666
I
II
III
IV
V
VI
h
Cluster Proportion
Cluster Labels
CK689
CK666
*

Figure S1. Subcellular protrusion phenotypes identified by the unsupervised teacher model. (**a**) The average Silhouette value and the number of clusters with the varying number of neighbors in the community detection clustering. (**b**) The t-SNE plot of the autocorrelation functions of protrusion velocity time series overlaid with cluster assignments. (**c**) The distance similarity heatmap ordered by the cluster labels. (**d**) The full protrusion velocity heatmaps in six identified phenotypes. (**e**) Averaged velocity time series in each cluster in CK689(control) and CK666-treated cells (m: the number of probing windows in CK689-treated cells; m': the number of probing windows in CK666-treated cells). (**f-g**) The t-SNE plot of ACFs overlaid with the data density and cluster assignments CK689 (f), CK666 (g). (**h**) Effects of CK666 on each protrusion phenotype. * $p<0.05$ indicates the statistical significance by bootstrap resampling. The numbers of cells: 10 for CK689 and 10 for CK666. (**i-j**) the t-SNE plot of ACFs overlaid with cluster assignments in DMSO (**i**) and blebbistatin-treated cells (**j**).

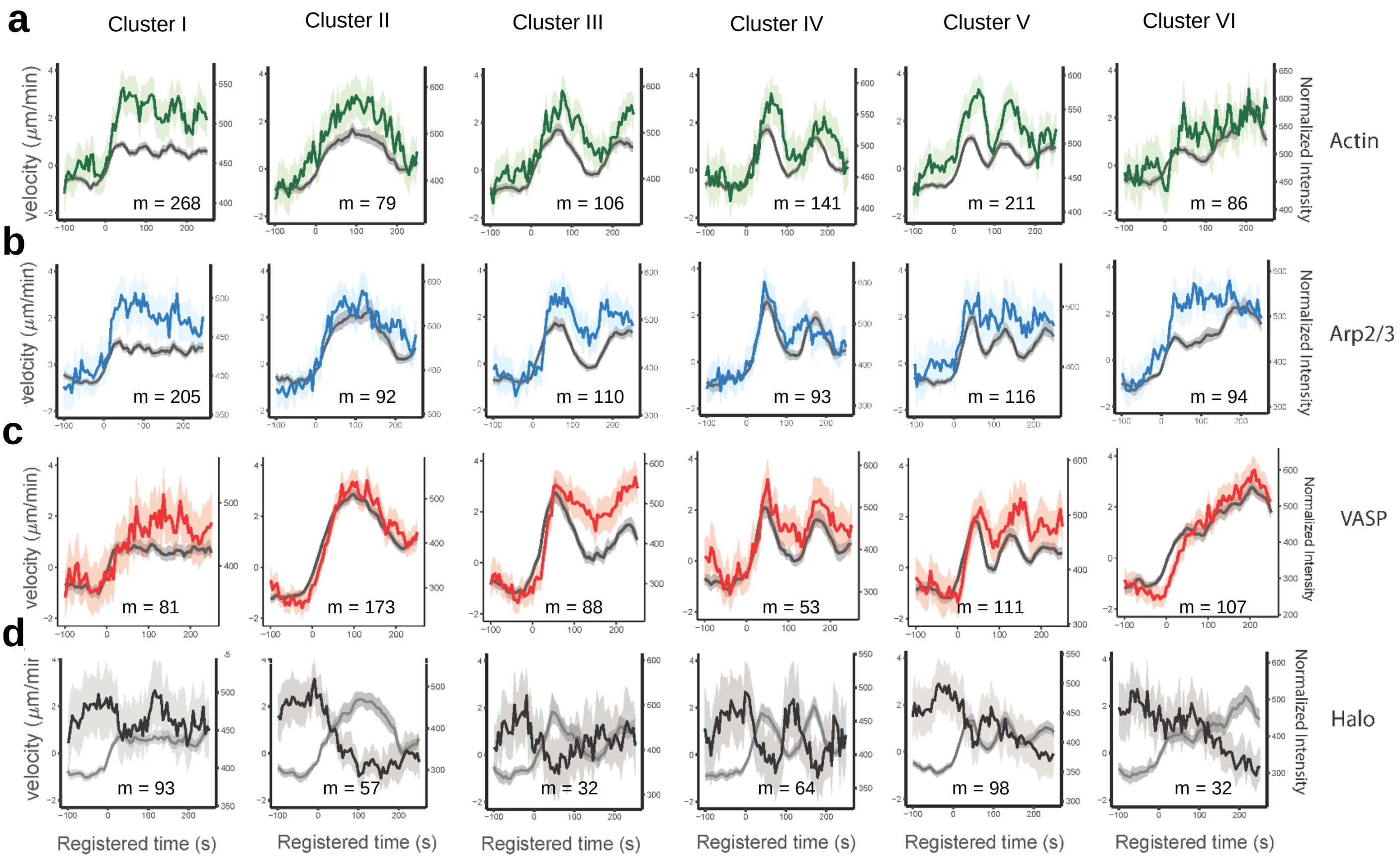

Figure S2. Differential actin regulator dynamics associated with subcellular protrusion phenotypes. (**a–d**) Averaged normalized fluorescence intensity time series in each phenotype, overlaid with the corresponding velocity profiles. Time series are registered with respect to protrusion onset (t = 0). Solid lines indicate population averages. Shaded error bands indicate 95% confidence intervals of the mean computed by bootstrap resampling. The gray lines indicate protrusion velocity time series associated with the indicated fluorescent proteins. m: the number of probing windows. The numbers of cells: 10 for actin, 11 for Arp2/3, 9 for VASP, and 5 for Halo)

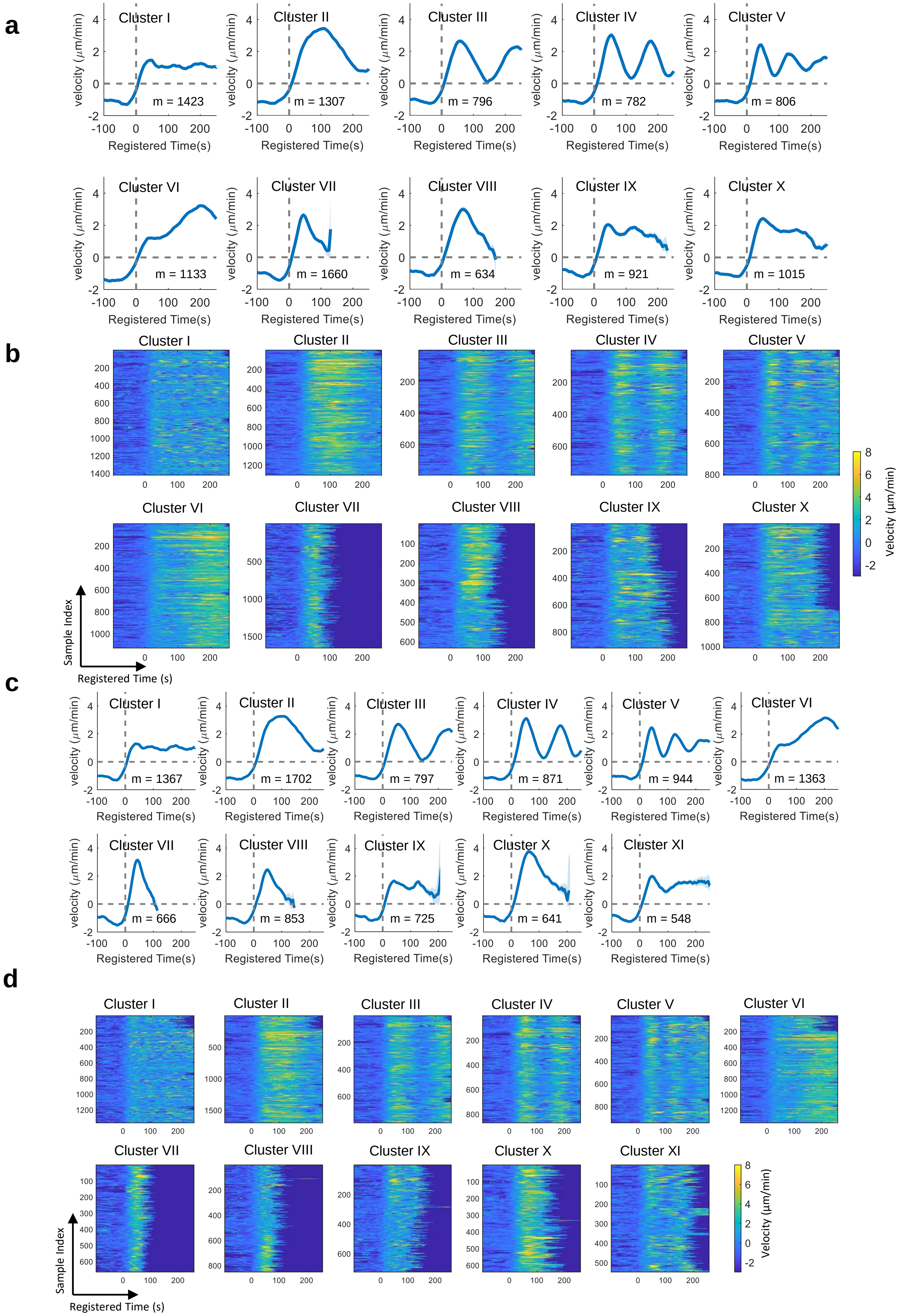
a
Cluster I
m = 1423
Cluster II
m = 1307
Cluster III
m = 796
Cluster IV
m = 782
Cluster V
m = 806
Cluster VI
m = 1133
Cluster VII
m = 1660
Cluster VIII
m = 634
Cluster IX
m = 921
Cluster X
m = 1015
velocity (μm/min)
Registered Time(s)
b
Cluster I
Cluster II
Cluster III
Cluster IV
Cluster V
Cluster VI
Cluster VII
Cluster VIII
Cluster IX
Cluster X
Velocity (μm/min)
Sample Index
Registered Time (s)
c
Cluster I
m = 1367
Cluster II
m = 1702
Cluster III
m = 797
Cluster IV
m = 871
Cluster V
m = 944
Cluster VI
m = 1363
Cluster VII
m = 666
Cluster VIII
m = 853
Cluster IX
m = 725
Cluster X
m = 641
Cluster XI
m = 548
velocity (μm/min)
Registered Time(s)
d
Cluster I
Cluster II
Cluster III
Cluster IV
Cluster V
Cluster VI
Cluster VII
Cluster VIII
Cluster IX
Cluster X
Cluster XI
Velocity (μm/min)
Sample Index
Registered Time (s)

Figure S3. Subcellular protrusion phenotypes from variable-length time series of protrusion velocities. (**a-b**) Averaged velocity time series profiles in each protrusion phenotype based on ACF features (**a**) and the corresponding velocity heatmaps (**b**). (**c-d**) Averaged velocity time series profiles in each protrusion phenotype based on DFs extracted by the student model (**c**) and the corresponding velocity heatmaps (**d**). Solid lines indicate population averages and shaded error bands indicate 95% confidence intervals of the mean estimated by bootstrap resampling. The error bars indicate 95% confidence interval of the mean of the cluster proportions. m: the number of probing windows.

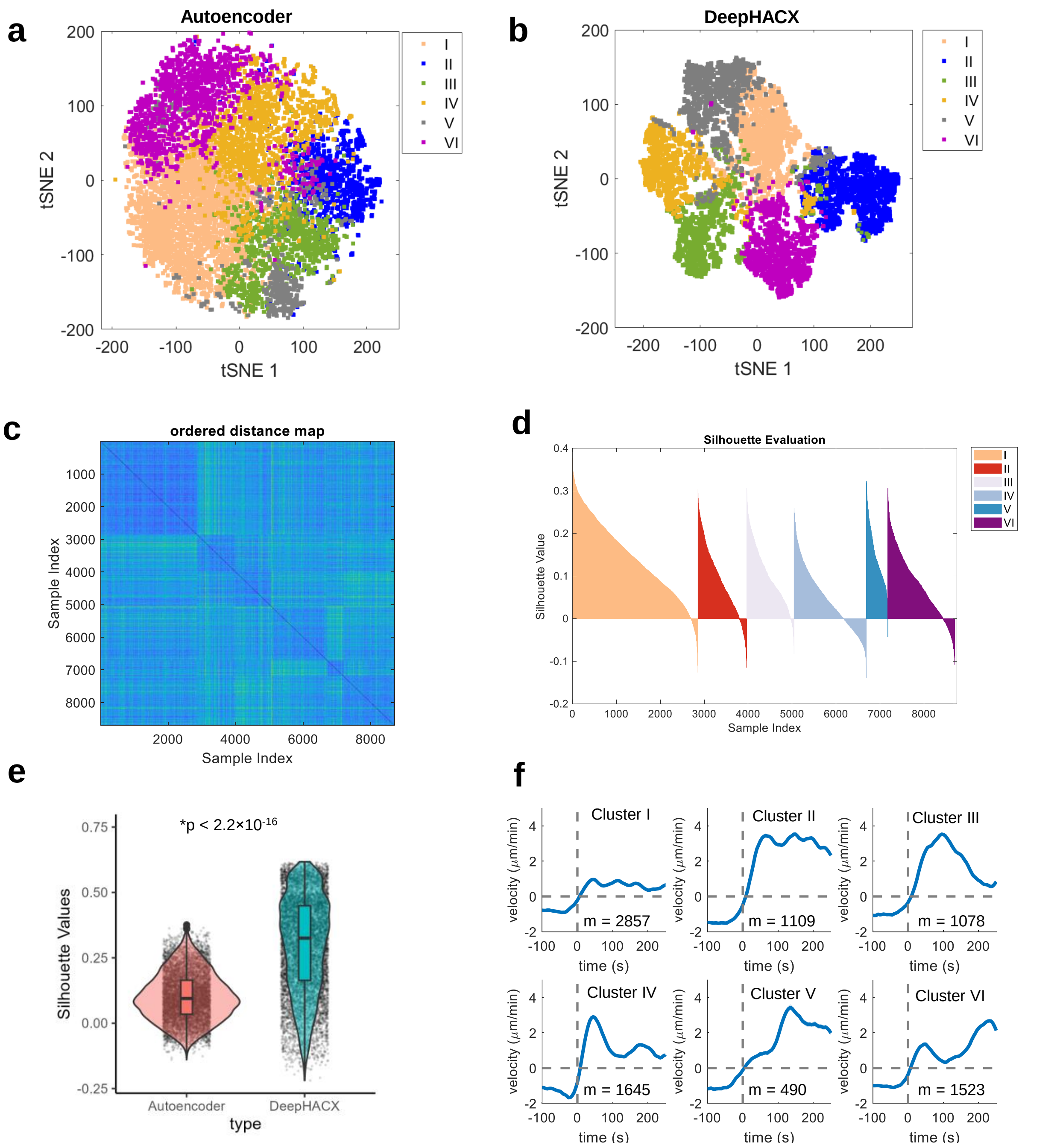

Figure S4. Subcellular protrusion phenotypes identified by deep features from the conventional autoencoder. (**a-b**) The t-SNE plots of the DFs of protrusion velocity time series with various length overlaid with cluster assignments using the conventional autoencoder (a) and DeepHACX (b). (**c**) The distance similarity heatmap ordered by the cluster labels. (**d**) Silhouette value per each sample across different clusters. (**e**) A comparison of the distributions of silhouette values with the conventional autoencoder and DeepHACX. * $p<2.2\times10^{-16}$ indicates the statistical significance by two-sided Wilcoxon rank sum test. The number of samples: 8702 (autoencoder, DeepHACX) (**f**) Averaged velocity time series profiles in each protrusion phenotype based on DFs extracted by the autoencoder model. Solid lines indicate population averages and shaded error bands indicate 95% confidence intervals of the mean estimated by bootstrap resampling. The *m* represents the number of probing windows.

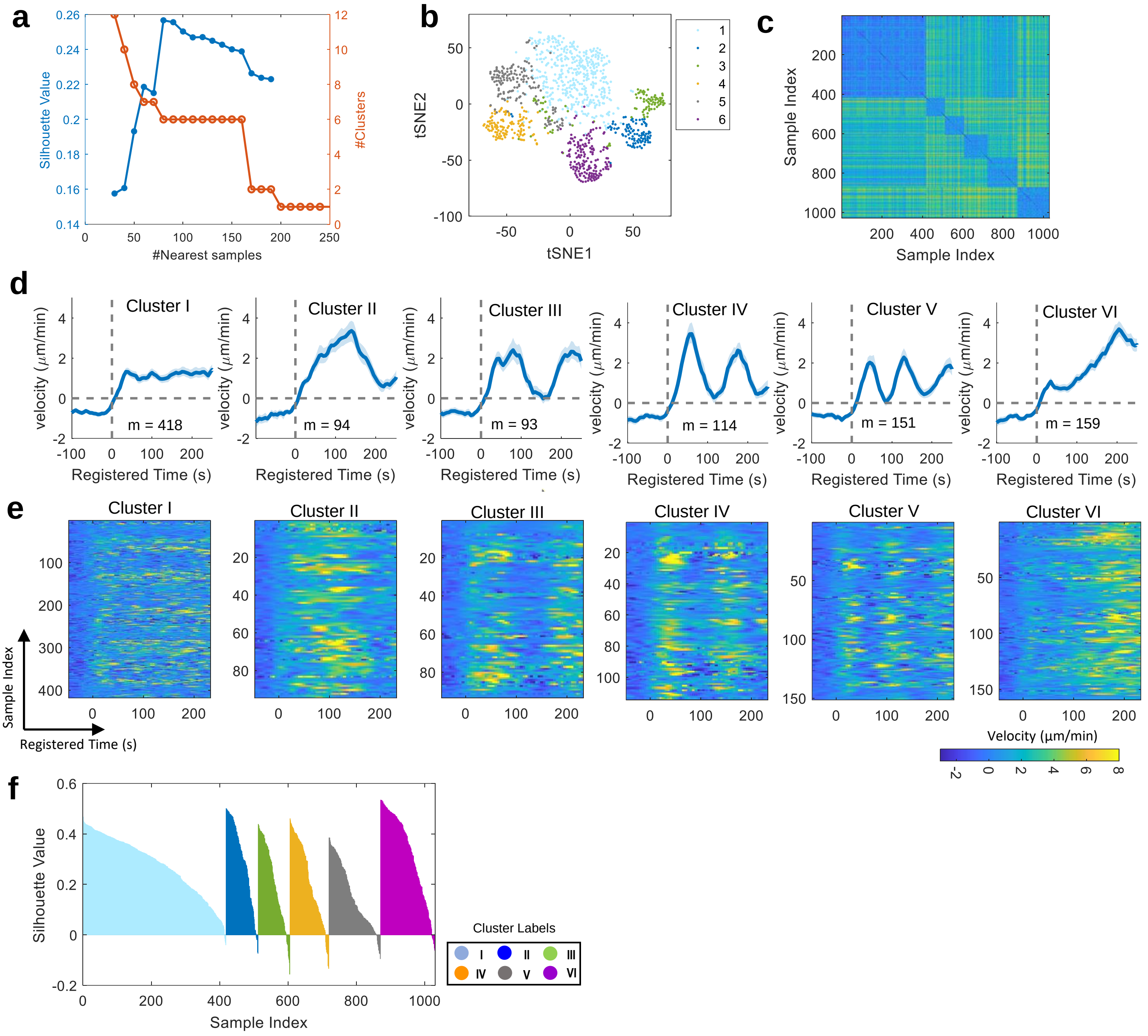

Figure S5. Deep feature-based Subcellular protrusion phenotypes of MCF10A cells. (a) The average Silhouette value and the number of clusters with the varying number of neighbors in the community detection clustering. (**b**) The t-SNE plot of the DFs of protrusion velocity time series overlaid with cluster assignments. (**c**) The distance similarity heatmap ordered by the cluster labels. (**d-e**) Averaged velocity time series profiles in each protrusion phenotype based on DFs extracted by the student model (**d**) and the corresponding velocity heatmaps (**e**). Solid lines indicate population averages and shaded error bands indicate 95% confidence intervals of the mean estimated by bootstrap resampling. **(f)** Silhouette value per each sample across different clusters. The *m* represents the number of probing windows. The number of MCF10A cells: 16.

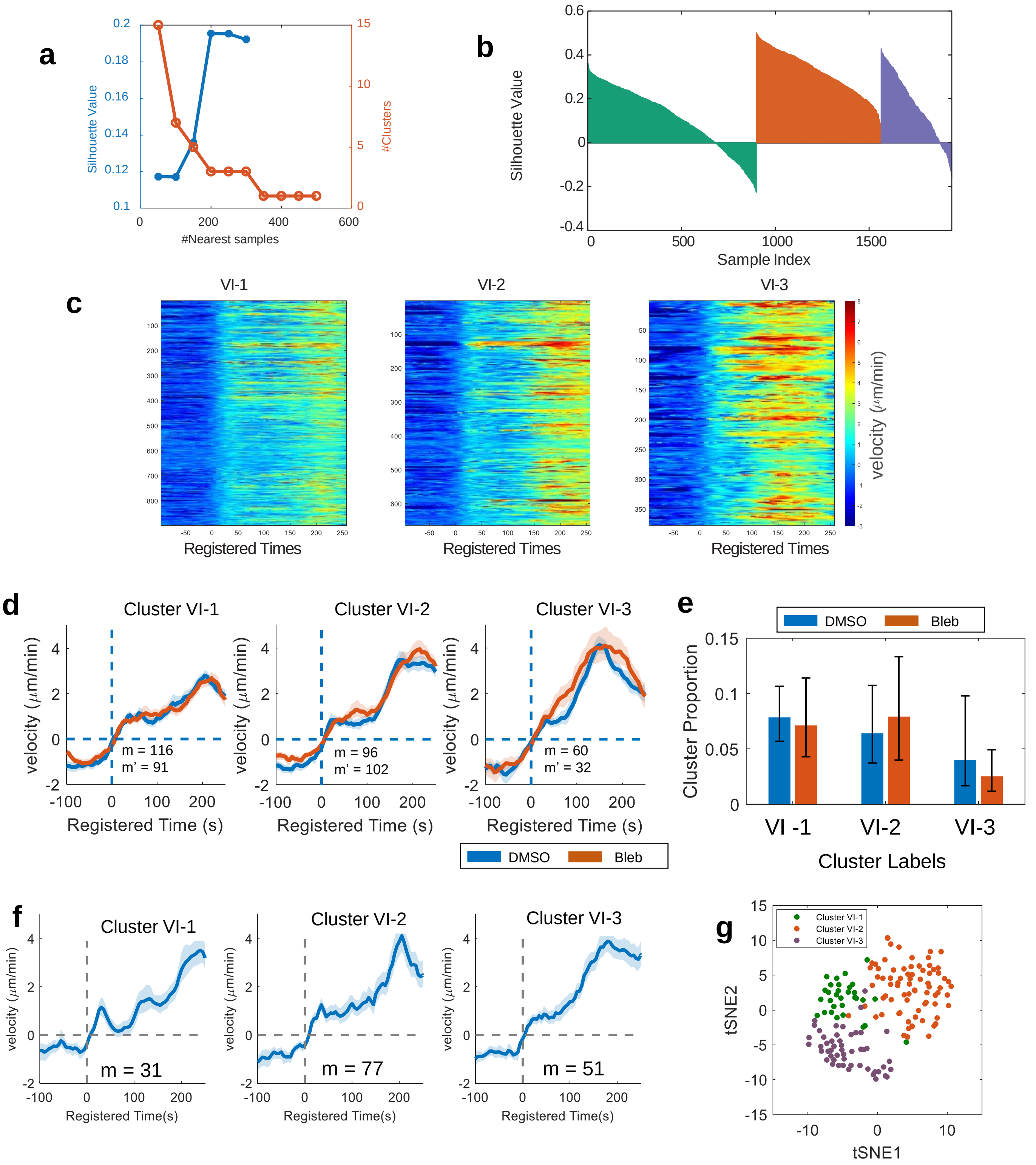

Figure S6. Sub-clustering results from accelerating protrusion phenotype. (**a**) The average Silhouette value and the number of clusters with the varying number of neighbors in the community detection clustering. (**b**) The Silhouette plot of the clustering result. (**c**) The velocity heatmap of three deep accelerating protrusion phenotypes. (**d**) Averaged velocity time series profiles in each deep phenotype of accelerating protrusion in DMSO and blebbistatin-treated cells (m: the number of probing windows in DMSO; m': the number of probing windows in blebbistatin-treated cells). (**e**) Effects of blebbistatin on each protrusion phenotype. There was no statistical significance in each phenotype. The numbers of cells: 14 for DMSO and blebbistatin for 13. The error bars indicate 95% confidence interval of the mean of the cluster proportions. (**f-g**) Sub-clustering results of the accelerating protrusion phenotype of MCF10A cells. (**f**) Averaged velocity time series profiles in each deep phenotype. m: the number of probing windows. (**g**) The t-SNE visualization of the DFs in accelerating protrusion phenotype. Solid lines indicate population averages and shaded error bands indicate 95% confidence intervals of the mean estimated by bootstrap resampling. The number of MCF10A cells: 16.

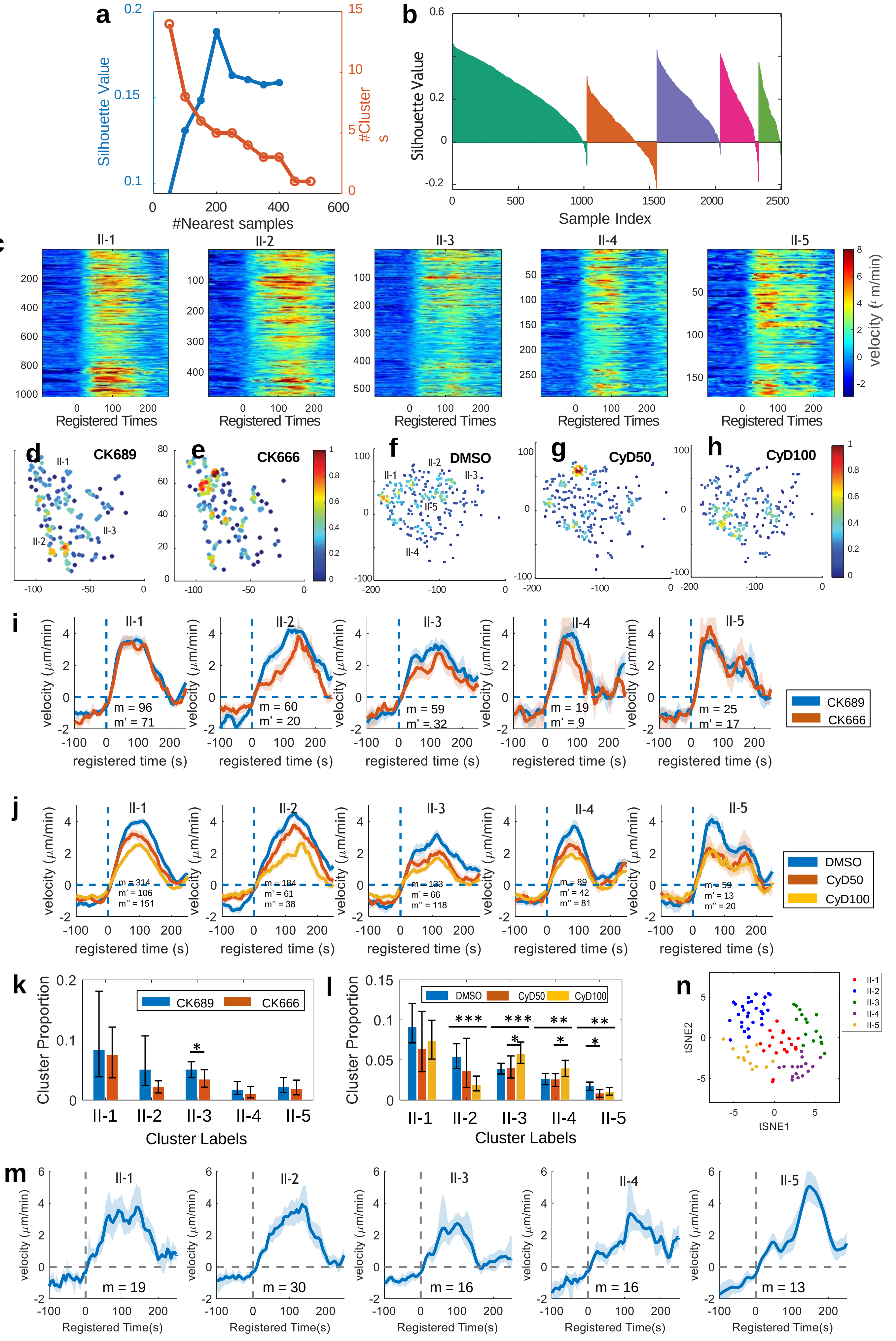
a
Silhouette Value
#Clusters
#Nearest samples
b
Silhouette Value
Sample Index
c
II-1
II-2
II-3
II-4
II-5
Registered Times
velocity (µ m/min)
d
CK689
e
CK666
f
DMSO
g
CyD50
h
CyD100
i
velocity (µm/min)
registered time (s)
m = 96
m' = 71
m = 60
m' = 20
m = 59
m' = 32
m = 19
m' = 9
m = 25
m' = 17
CK689
CK666
j
m = 314
m' = 106
m'' = 151
m = 184
m' = 61
m'' = 38
m = 133
m' = 66
m'' = 118
m = 89
m' = 42
m'' = 81
m = 59
m' = 13
m'' = 20
DMSO
CyD50
CyD100
k
Cluster Proportion
Cluster Labels
l
n
tSNE1
tSNE2
m
Registered Time(s)
m = 19
m = 30
m = 16
m = 16
m = 13

Figure S7. Sub-clustering results from bursting protrusion phenotype. (**a**) The average Silhouette value and the number of clusters with the varying number of neighbors in the community detection clustering. (**b**) The Silhouette plot of the clustering result. (**c**) The velocity heatmap of three deep bursting protrusion phenotypes. (**d-f**) The t-SNE visualization of the deep features of the bursting protrusion for CK666 (**d-e**) and Cytochalasin D (**f-h**) treated cells. The color indicates the density of data on the t-SNE plots. (**i-j**) Averaged velocity time series profiles in each deep bursting protrusion phenotype in CK689/CK666-treated cells (**i**) (m: the number of probing windows in CK689-treated cells; m': the number of probing windows in CK666-treated cells). and DMSO/Cytochalasin D-treated cells (**j**) (m: the number of probing windows in DMSO-treated cells; m': the number of probing windows in CyD50-treated cells; m'': the number of probing windows in CyD100-treated cells). (**k-l**) Effects of CK666 (**k**) and Cytochalasin D (**l**) on each protrusion phenotype. The error bars indicate 95% confidence interval of the mean of the phenotype proportions. *$p < 0.05$, ** $p < 0.01$, *** $p < 0.001$ indicate the statistical significance by bootstrap sampling. The numbers of cells: 10 for CK689, 10 for CK666 (i-k) and 22 for DMSO, 16 for CyD50, 20 for CyD100 (j-l). (**m**-**n**) Sub-clustering results of the bursting protrusion phenotype of MCF10A cells. (**m**) Averaged velocity time series profiles in each deep phenotype. m: the number of probing windows. (**n**) The t-SNE visualization of the deep features in burstng protrusion phenotype. Solid lines indicate population averages and shaded error bands indicate 95% confidence intervals of the mean estimated by bootstrap sampling. The number of MCF10A cells: 16.

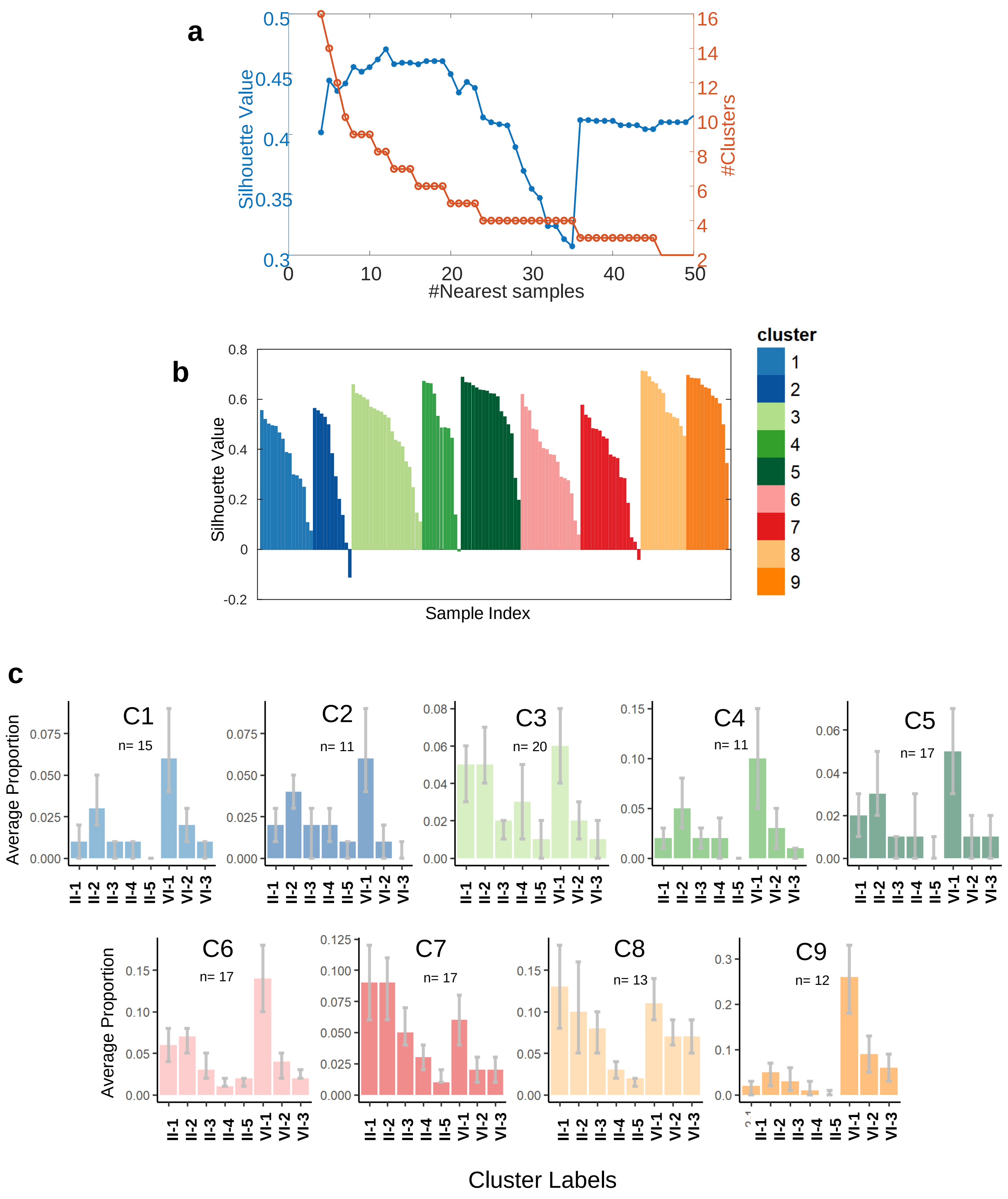

Figure S8. Cellular protrusion phenotyping. (**a**) The average Silhouette values and the number of clusters with the varying number of neighbors in the community detection clustering. (**b**) The Silhouette plot of the clustering result. (**c**) The proportions of deep phenotypes in each cell phenotypes (n: the number of cells in each cell cluster). The 95% confidence intervals of the mean were estimated by bootstrap sampling. The total number of cells: 133.

Table S1. Statistical testing of clusters using ACF features on CK689/CK666 experiments (Supplementary Fig. 1h).

	Cluster 1	Cluster 2	Cluster 3	Cluster 4	Cluster 5	Cluster 6
P value	0.0447	0.2060	0.0632	0.3748	0.4583	0.0127
Effect Size	2.4070	1.1614	2.1164	0.4708	0.1360	3.0357

Table S2. Statistical testing of clusters using ACF features on DMSO/blebbistatin experiments (Fig. 2f).

	Cluster 1	Cluster 2	Cluster 3	Cluster 4	Cluster 5	Cluster 6
P value	0.0159	0.0038	0.2878	0.3673	0.1980	0.3941
Effect Size	2.9296	3.4723	0.7560	0.4547	1.1889	0.3930

Table S3. The details of the model structure and setting hyper-parameters.

Parameters	Values
**Encoder**	Input (1) -> Bi-LSTM(10) -> LSTM(20) -> LSTM(30)
**Regularize**	Dense (32) -> BatchNormalization -> *Relu* activation -> Dropout (0.5) -> Dense -> *softmax* Activation
**Decoder**	Bi-LSTM(30) -> Bi-LSTM(20) -> LSTM(1)
**Activation function**	tanh (LSTM / Bi-LSTM)
**Optimizer**	rmsprop
**Loss functions**	categorical crossentropy (***Classifer***), mean squared error (***Encoder***)
**Metrics**	mean squared error
**Loss weight**	1, 25
**Batch size**	128
**ratio (train: validation: test)**	0.49, 0.21, 0.3
**Max interactions**	25000
**Epochs**	237
**Learning rate**	default value (0.001)
**Dataset Size**	27540

Table S4. Statistical testing of clusters using deep features on CK689/CK666 experiments (Fig. 4d).

	Cluster 1	Cluster 2	Cluster 3	Cluster 4	Cluster 5	Cluster 6
P value	0.0160	0.1210	0.0884	0.2797	0.2593	0.0069
Effect Size	2.9415	1.6765	1.8965	0.7524	0.9192	3.4520

Table S5. Statistical testing of clusters using deep features on DMSO/CyD experiments (Fig. 4i).

		Cluster 1	Cluster 2	Cluster 3	Cluster 4	Cluster 5	Cluster 6
P value	DMSO vs CyD50	0.0213	0.0707	0.4484	0.2829	0.0512	0.0272
	DMSO vs CyD100	0.0228	0.1455	0.2659	0.0763	0.4602	0.0132
	CyD50 vs CyD100	0.2911	0.2659	0.2446	0.0563	0.0709	0.3743
Effect Size	DMSO vs CyD50	2.7469	2.0811	0.1799	0.7783	2.2609	2.7143
	DMSO vs CyD100	2.8425	1.5027	0.8920	1.9855	0.1551	3.2831
	CyD50 vs CyD100	0.7999	0.9135	0.9785	2.2449	2.0333	0.4312

Table S6. Statistical testing of clusters using deep features on DMSO/blebbistatin experiments (Fig. 4m).

	Cluster 1	Cluster 2	Cluster 3	Cluster 4	Cluster 5	Cluster 6
P value	0.0100	0.0004	0.3381	0.4480	0.1466	0.4577
Effect Size	3.1741	3.8240	0.5987	0.2186	1.4813	0.1789

Table S7. Statistical testing of fine-grained accelerating protrusion clusters using deep features on CK689/CK666 experiments (Fig. 5d).

	Cluster VI-1	Cluster VI-2	Cluster VI-3
P value	0.0668	0.0094	0.0003
Effect Size	2.1546	3.0094	4.4226

Table S8. Statistical testing of fine-grained accelerating protrusion clusters using deep features on DMSO/CyD experiments (Fig. 5f).

		Cluster VI-1	Cluster VI-2	Cluster VI-3
P value	DMSO vs CyD50	0.0853	0.4717	0.0221
	DMSO vs CyD100	0.3207	0.0135	0.0008
	CyD50 vs CyD100	0.1835	0.0509	0.2473
Effect Size	DMSO vs CyD50	1.9764	0.0609	2.8304
	DMSO vs CyD100	0.6463	3.2407	4.0853
	CyD50 vs CyD100	1.2956	2.3109	0.9918

Table S9. Statistical testing of fine-grained accelerating protrusion clusters using deep features on DMSO/blebbistatin experiments (Fig. S6e).

	Cluster VI-1	Cluster VI-2	Cluster VI-3
P value	0.4471	0.3104	0.3198
Effect Size	0.1943	0.7027	0.7796

Table S10 Statistical testing of fine-grained bursting protrusion clusters using deep features on CK689/CK666 experiments (Fig. S7k).

	Cluster II-1	Cluster II-2	Cluster II-3	Cluster II-4	Cluster II-5
P value	0.4546	0.0595	0.0358	0.1928	0.2074
Effect Size	0.2430	2.0018	2.4870	1.2088	1.1284

Table S11 Statistical testing of fine-grained bursting protrusion clusters using deep features on DMSO/CyD experiments (Fig. S7l).

		Cluster II-1	Cluster II-2	Cluster II-3	Cluster II-4	Cluster II-5
P value	DMSO vs CyD50	0.1058	0.1638	0.1906	0.3411	0.0032
	DMSO vs CyD100	0.1405	0.0003	<0.001	0.0077	0.0375
	CyD50 vs CyD100	0.3386	0.1032	0.0126	0.0396	0.1512
Effect Size	DMSO vs CyD50	1.7812	1.4069	1.2363	0.5794	4.0007
	DMSO vs CyD100	1.5249	5.2781	4.9689	3.2464	2.5609
	CyD50 vs CyD100	0.5593	1.7371	3.0703	2.4716	1.4420

Table S12. Statistical testing of fine-grained bursting protrusion clusters using deep features on DMSO/Blebbistatin experiments (Fig. 5j).

	Cluster II-1	Cluster II-2	Cluster II-3	Cluster II-4	Cluster II-5
P value	<0.001	0.1903	0.1402	0.2393	0.1146
Effect Size	4.3205	1.2417	1.4986	0.9820	1.6793

Table S13. The numbers of cells in Cell Clusters

Cell Cluster	Total	1	2	3	4	5	6	7	8	9
Number of cells	133	15	11	20	11	17	17	17	13	12

Table S14. Summary of single-cell protrusion phenotypes of PtK1 cells

Cell Cluster #	Dominant Protrusion	Cell Phenotype	Drug-Sensitive Deep Phenotypes	
			Cluster II-1 (blebbistatin)	Cluster VI-2/3 (CK666/CyD)
1	Steady	Steady Cell1	Low	Low
2	Steady	Steady Cell2	Low	Low
3	Steady & Periodic #1	Periodic Cell1	**Mid**	Low
4	Steady & Periodic #2	Periodic Cell2	Low	Low
5	Steady & Periodic #3	Periodic Cell3	Low	Low
6	Steady, Bursting, & Accelerating	Mid-Bursting/ Accelerating Cell	**Mid**	**Mid**
7	Bursting	Bursting Cell	**High**	Low
8	Bursting & Accelerating	Strong-Bursting/ Accelerating Cell	**High**	**High**
9	Accelerating	Accelerating Cell	Low	**High**

Table S15. Statistical testing of the proportional differences between Bursting and Steady Cell Groups, and Accelerating and Steady Cell Groups in three paired experiments (Fig. 7f-g-h).

	CK689/CK666		DMSO/CyD50+100		DMSO/bleb	
Proportional differences	Bursting -Steady	Acc-Steady	Bursting-Steady	Acc-Steady	Bursting-Steady	Acc-Steady
P value	0.123	0.014	<0.001	0.005	0.001	<0.001
